# Implementation of tenecteplase for acute ischemic stroke: workflow and safety in routine care including off-label use

**DOI:** 10.3389/fneur.2026.1882561

**Published:** 2026-10-28

**Authors:** Alexander Sekita, Kosmas Macha, Matthias C. Borutta, Maximilian I. Sprügel, Ludwig Singer, Tobias Engelhorn, Lena Mers, Anna Bogdanova, Stefan Schwab, Stefan T. Gerner

**Affiliations:** 1 Department of Neurology, University Hospital Erlangen, Erlangen, Germany; 2 Department of Neuroradiology, University Hospital Erlangen, Erlangen, Germany

**Keywords:** alteplase, intravenous thrombolysis, ischemic stroke, off label, procedure times, safety, tenecteplase

## Abstract

**Introduction:**

Tenecteplase is increasingly adopted as an alternative to alteplase for intravenous thrombolysis in acute ischemic stroke. However, real-world data describing complete institutional transitions, particularly in extended treatment windows and off-label clinical scenarios, remain limited.

**Methods:**

We conducted a retrospective cohort study of consecutive patients treated with intravenous thrombolysis during the 12 months before and after a complete institutional transition from alteplase to tenecteplase (June 2023–May 2025). Primary endpoints were in-hospital workflow metrics (door-to-needle, door-to-imaging, imaging-to-needle, door-to-groin, and door-to-recanalization times). Safety outcomes included any intracranial hemorrhage and symptomatic intracranial hemorrhage according to the SITS-MOST definition. Outcomes were analyzed in the overall cohort, in patients treated beyond 4.5 h after symptom onset, and in patients receiving oral anticoagulation. Multivariable-adjusted and propensity score–based analyses, including augmented inverse probability of treatment weighting, were applied to account for baseline differences.

**Results:**

Among 591 thrombolyzed patients (rt-PA *n* = 278; TNK *n* = 313), 31% were treated beyond 4.5 h and 14% under oral anticoagulation. Tenecteplase was associated with significantly shorter door-to-needle times (31 vs. 35 min; *p* = 0.004), primarily driven by reduced imaging-to-needle intervals (13 vs. 16 min; *p* = 0.004). Door-to-groin times were numerically shorter with tenecteplase but did not differ significantly (70 vs. 76 min; *p* = 0.07). Rates of any intracranial hemorrhage (8% vs. 7%; *p* = 0.61) and symptomatic intracranial hemorrhage (3% vs. 1%; *p* = 0.34) were comparable. Similar safety findings were observed in patients treated beyond 4.5 h after symptom onset and in those receiving oral anticoagulation, with consistent results across multivariable-adjusted and propensity score–based analyses.

**Conclusion:**

Institutional transition to tenecteplase was associated with improved in-hospital workflow without evidence of increased hemorrhagic complications. These findings support the feasibility of implementing tenecteplase as the standard thrombolytic agent in routine comprehensive stroke center practice, including in extended treatment windows and selected off-label clinical scenarios.

## Introduction

Intravenous thrombolysis (IVT) remains the cornerstone in the treatment of acute ischemic stroke (AIS), with alteplase (rt-PA) serving as the standard of care for more than two decades (1). Tenecteplase (TNK), a genetically modified tissue plasminogen activator, has emerged as an attractive alternative. Due to its greater fibrin specificity, longer half-life and single-bolus administration, TNK offers practical advantages that may simplify treatment workflows and reduce treatment delays, particularly in high-volume stroke centers (2).

Several randomized controlled trials have established the non-inferiority of TNK compared with rt-PA within the conventional 4.5-h treatment window as well as in patients eligible for mechanical thrombectomy (3–6). More recently, the evidence base has expanded to include patients treated beyond 4.5 h after symptom onset. Trials such as TIMELESS, TRACE-III, OPTION, and CHABLIS-T II used perfusion imaging to select patients with salvageable tissue, however, the findings across these studies have been heterogeneous. While TRACE-III and OPTION reported improved functional outcomes with TNK compared with standard medical therapy in patients without access to thrombectomy, TIMELESS and CHABLIS-T II did not demonstrate superiority of TNK over placebo or best medical therapy in otherwise comparable, imaging-selected cohorts with high rates of large/medium vessel occlusion and thrombectomy eligibility (7–10).

These discrepant results, together with the current regulatory restriction of TNK approval to the 4.5-h window and practical concerns about dosing errors when multiple thrombolytic agents are used in parallel, may contribute to ongoing reluctance towards a full transition from rt-PA to TNK in routine clinical practice (2, 11). Importantly, a considerable proportion of real-world stroke patients present in extended time windows or under clinical constellations requiring particular consideration, including oral anticoagulation (OAC) and advanced age. Although intravenous alteplase has demonstrated benefit also in patients aged >80 years, evidence specifically addressing the safety and efficacy of TNK in this age group remains comparatively limited (12, 13). Such populations remain under-represented in randomized trials. Consequently, the real-world implications of institutional implementation of TNK across the full spectrum of stroke presentations remain insufficiently characterized, and pragmatic implementation data are needed to inform clinical practice (14, 15).

In June 2024, our tertiary comprehensive stroke center implemented a full institutional transition from rt-PA to TNK as the standard thrombolytic agent. This setting provided the opportunity to evaluate the real-world implementation of TNK and its impact on in-hospital workflow and safety outcomes in an unselected consecutive cohort treated in routine clinical practice, including patients presenting in extended treatment windows and selected higher-risk clinical scenarios.

## Methods

### Study design and patient population

This single-center observational cohort study was conducted at a tertiary comprehensive stroke center and was based on prospectively collected data from the institutional stroke registry. All consecutive patients treated with IVT, with or without subsequent endovascular thrombectomy (EVT), between June 1, 2023 and May 31, 2025 were eligible for inclusion. In June 2024, TNK replaced rt-PA as the standard thrombolytic agent according to our institutional standard operating procedure (SOP). The institutional transition was performed in a structured manner, including adaptation of the institutional SOP and dedicated staff training prior to implementation. A detailed description of the implementation process, training procedures, and institutional transition workflow is provided in the Supplementary Methods. Patients treated during the 12 months prior to this transition (June 2023–May 2024) formed the rt-PA cohort, whereas those treated during the subsequent 12 months (June 2024–May 2025) constituted the TNK cohort. The first six months after the institutional transition have been previously reported as an initial institutional experience focusing primarily on feasibility and early workflow effects (16). The present analysis represents a substantially expanded cohort including the full 24-month observation period as well as additional safety analyses and propensity score–based adjustment approaches that were not performed in the pilot report.

Apart from the replacement of rt-PA with TNK as the standard thrombolytic agent, no structural modifications to stroke workflow pathways, imaging protocols, or staffing structures were implemented during the study period. The institutional SOP was updated to incorporate TNK administration and dosing prior to implementation. In preparation for the transition, standardized on-site training sessions were conducted for treating physicians and stroke nurses to ensure protocol adherence and safe administration of TNK.

Patients undergoing EVT without preceding IVT were excluded. In addition, 21 patients with secondary neurological deterioration who received IVT only after deterioration during hospitalization were excluded. All other consecutive IVT-treated patients were included irrespective of treatment time window or clinical risk profile in order to reflect routine comprehensive stroke center practice.

### Acute stroke management

Both rt-PA (Boehringer Ingelheim; 0.9 mg/kg, maximum 90 mg) and TNK (Boehringer Ingelheim; 0.25 mg/kg, maximum 25 mg) were administered according to current guideline recommendations and at the discretion of the treating physician. Rt-PA was given as an initial bolus followed by a one-hour continuous infusion, whereas TNK was administered as a single intravenous bolus.

Initial brain imaging consisted of non-contrast computed tomography (CT; Siemens SOMATOM X.ceed) or magnetic resonance imaging (MRI; Siemens MAGNETOM Sola Fit 1.5 T). IVT decisions followed institutional SOPs and guideline recommendations, with non-contrast CT as the primary imaging modality for patients presenting within 4.5 h of symptom onset and IVT initiation directly after imaging when appropriate. Multimodal imaging was additionally performed at the discretion of the treating physician in cases of unclear or unknown symptom onset, suspected large vessel occlusion (LVO), or when further tissue-based assessment was required to support treatment decisions, including selected patients presenting within the conventional time window. According to our institutional SOP, which has been described previously, patients presenting in the extended 4.5–24-h window underwent multimodal imaging with CT or MRI perfusion to identify a perfusion–core mismatch indicating salvageable tissue and to guide off-label IVT and reperfusion therapy in selected cases without advanced established infarction (16).

In patients receiving OAC, off-label IVT decisions were assessed individually. Treatment was permitted for vitamin K antagonist users if the international normalized ratio (INR) was ≤ 1.7. For patients on direct oral anticoagulants, IVT was considered if plasma drug concentrations were below defined thresholds (≤ 100 ng/mL for factor-Xa inhibitors measured by anti-Xa activity; ≤ 100 ng/mL for dabigatran measured by Hemoclot assay).

Routine follow-up imaging was performed 24 h after IVT or earlier in cases of neurological worsening to assess for intracranial hemorrhage (ICH). For patients undergoing EVT, angiographic reperfusion success was evaluated by a neuroradiologist using the modified Thrombolysis in Cerebral Infarction (mTICI) scale, with scores ≥ 2b considered successful. Neurological severity was assessed using the National Institutes of Health Stroke Scale (NIHSS) at admission, after 24 and 72 h, and at discharge.

### Outcome measures

The primary endpoint was in-hospital treatment efficiency, quantified by door-to-needle (DTN), door-to-imaging (DTI), imaging-to-needle (ITN), door-to-groin (DTG), and door-to-recanalization (DTR) times. The time of arrival in the imaging suite (CT or MRI) was used to calculate DTI and ITN intervals. Secondary outcomes included early neurological changes and exploratory in-hospital clinical outcomes at discharge. Neurological severity was assessed using the NIHSS at admission, 24 h, 72 h, and discharge to evaluate changes over time. Early major neurological improvement was defined as a decrease of at least 4 NIHSS points within 72 h, and early neurological deterioration as an increase of at least 4 NIHSS points over the same period. Functional outcome at discharge was analyzed as an exploratory in-hospital measure using standard dichotomized definitions of excellent outcome (mRS 0–1) and favorable functional outcome (mRS 0–2). The full distribution of mRS scores at discharge was also examined as an exploratory outcome.

Safety endpoints included any ICH and symptomatic ICH, primarily assessed according to the Safe Implementation of Thrombolysis in Stroke – Monitoring Study (SITS-MOST) definition, with additional evaluation using European Cooperative Acute Stroke Study II (ECASS-II) and National Institute of Neurological Disorders and Stroke (NINDS) criteria for enhanced analysis (17–19). Further safety outcomes comprised parenchymal hematoma type 2 (PH2) per Heidelberg Bleeding Classification and post-thrombolytic angioedema, defined as acute submucosal or subcutaneous swelling in the orofacial region or upper airway confirmed clinically (20, 21).

### Statistical analyses

Analyses were performed using SPSS Statistics version 28.0 (IBM Corp., United States), Stata version 18.5 (StataCorp, College Station, TX, United States) and R 4.5 (R Foundation for Statistical Computing, Vienna, Austria). Continuous variables are presented as medians with interquartile ranges and categorical variables as counts and percentages. Unmatched comparisons used the Mann–Whitney U test for continuous variables and Pearson’s chi-square or Fisher’s exact test for categorical variables. Associations between thrombolytic treatment and hemorrhagic outcomes were additionally evaluated using unadjusted and multivariable logistic regression. Treatment effects are reported as odds ratios and absolute risk differences with 95% confidence intervals; adjusted risk differences were obtained by marginal standardization.

Propensity scores were estimated using age, hypertension, CHA₂DS₂-VASc score, and treatment within 4.5 h. One-to-one nearest-neighbor matching without replacement was performed on the logit propensity score using a caliper of 0.2 standard deviations. Balance was assessed using absolute standardized mean differences (SMD), with values below 0.1 indicating acceptable balance (for further details see Supplementary Methods). Analyses of the matched cohort accounted for pairing: binary outcomes were compared using exact McNemar tests, continuous outcomes using paired Wilcoxon signed-rank tests, and the complete discharge mRS and discharge-destination distributions using Stuart–Maxwell tests. Matched risk differences were estimated with pair-clustered robust variance.

Augmented inverse probability of treatment weighting (AIPTW) was used to estimate the average treatment effect on the treated in the full cohort for any ICH and symptomatic ICH according to SITS-MOST. Subgroup analyses examined treatment time window, OAC, LVO, stroke severity, and age >80 years. Model specifications, matching procedures, outcome-specific tests, and effect estimators are detailed in the Supplementary Methods.

All tests were two-sided, with *p* < 0.05 considered statistically significant. No formal sample size calculation was performed; the sample comprised consecutive eligible patients during the study period. The study is reported in accordance with STROBE.

### Ethical approval

This study was conducted following the approval of the local ethics committee, FAU Erlangen-Nuremberg, Germany. The ethics vote number 377_17 Bc was obtained for the project titled "Clinical follow-up of patients with acute stroke" on 25 May 2020.

## Results

### Study population

Between 1 June 2023 and 31 May 2025, 796 patients with suspected AIS underwent recanalization therapy. After exclusion of 184 patients treated with EVT alone and 21 patients treated after secondary deterioration, 591 IVT-treated patients were included in the final analysis (Figure 1). Of these, 313 (53.0%) patients were treated with TNK and 278 (47.0%) with rt-PA.

**Figure 1:**
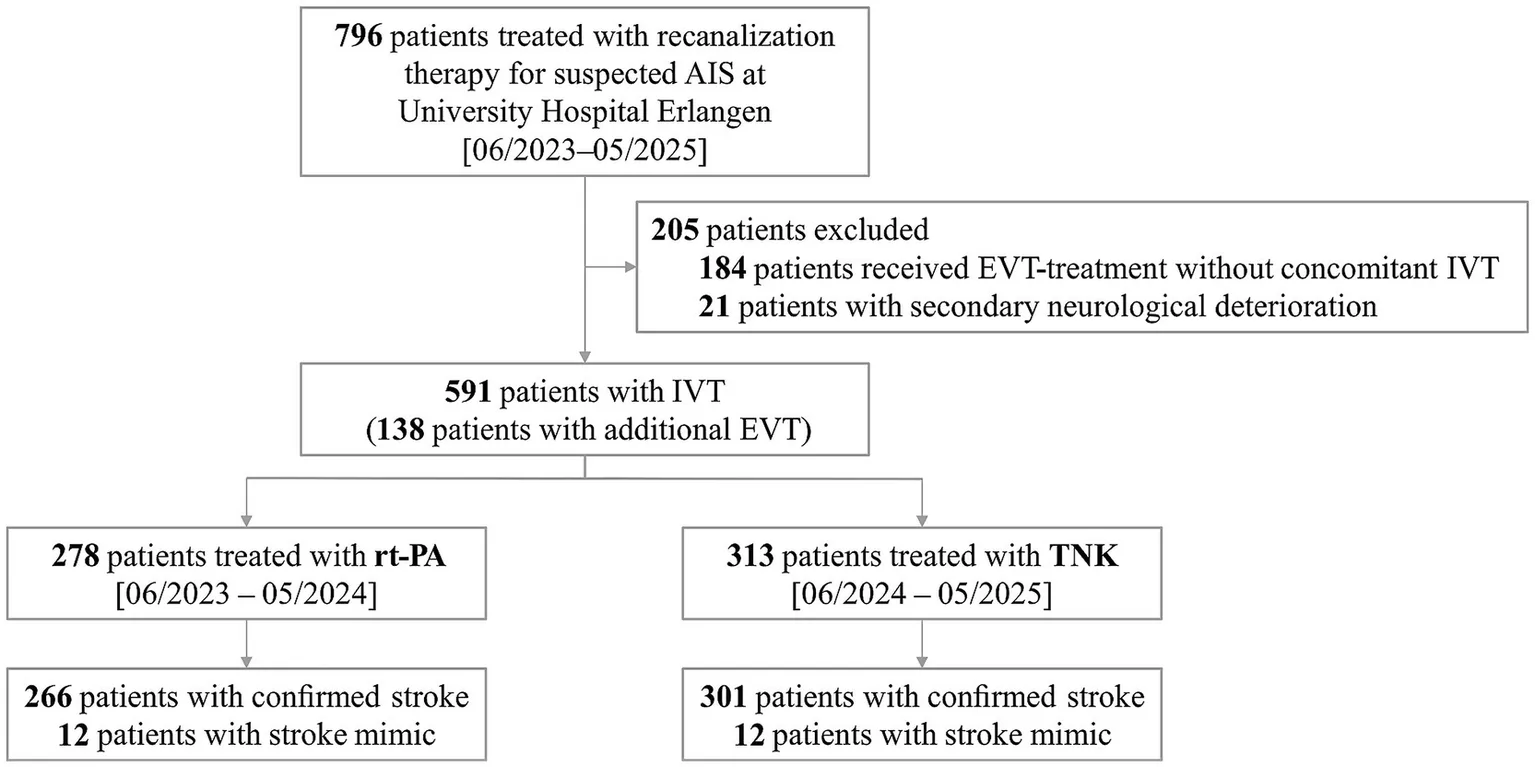
Flowchart of patient selection. Overall, 796 patients with suspected acute ischemic stroke underwent recanalization therapy over 24 months (June 2023–May 2025). Of those, 184 patients were treated with EVT alone and 21 were treated after secondary deterioration. After exclusion of those patients, 591 received IVT and were suitable for final analyses. AIS, acute ischemic stroke; EVT, endovascular treatment; IVT, intravenous thrombolysis.

### Baseline characteristics

Baseline demographic and clinical characteristics are summarized in Table 1. Patients treated with rt-PA had a higher prevalence of arterial hypertension and a higher median CHA₂DS₂-VASc score, whereas age, sex distribution, premorbid functional status, baseline NIHSS, cardiovascular risk factors, and prior antithrombotic medication were comparable between groups. OAC was present in 14% of TNK-treated and 15% of rt-PA–treated patients.

**Table 1:** Baseline characteristics for TNK and rt-PA cohorts.

IVT-treated patients (*n* = 591)	TNK (*n* = 313)	rt-PA (*n* = 278)	*p* value
Age, y; median (IQR)	75 (63–84)	77 (66–85)	0.09
Patients aged >80 years; *n* (%)	112 (36%)	107 (38%)	0.50
Female sex; *n* (%)	143 (46%)	135 (49%)	0.49
Prior medical history; *n* (%)			
Premorbid mRS; median (IQR)	1 (0–2)	1 (0–3)	0.28
Premorbid mRS 0	149 (48%)	129 (46%)	0.77
Heart failure	31 (10%)	32 (12%)	0.53
Hypertension	221 (71%)	228 (82%)	0.001
Diabetes mellitus type II	83 (27%)	71 (26%)	0.79
Previous Stroke or TIA	73 (23%)	74 (27%)	0.36
Vascular diseases^*^	49 (16%)	56 (20%)	0.15
Atrial fibrillation	44 (14%)	50 (18%)	0.19
CHA2DS2-VASc-score; median (IQR)	3 (2–5)	4 (2–5)	0.017
Comedication; *n* (%)			
OAC intake	43 (14%)	42 (15%)	0.64
DOAC	39 (91%)	34 (81%)	0.20
Vitamin K antagonist	4 (9%)	8 (19%)	
Any prior antiplatelet therapy	107 (34%)	99 (36%)	0.72
Single antiplatelet therapy	102 (33%)	97 (35%)	0.55
Dual antiplatelet therapy	5 (2%)	2 (1%)	0.33
Stroke characteristics			
NIHSS on admission; median (IQR)	6 (3–11)	6 (4–11)	0.42
Stroke severity; *n* (%)			0.06
NIHSS 0	13 (4%)	2 (1%)	
Mild (NIHSS 1–4)	101 (32%)	92 (33%)	
Moderate (NIHSS 5–15)	161 (51%)	143 (51%)	
Severe (NIHSS ≥16)	38 (12%)	41 (15%)	
Symptom-onset-to-door, min; median (IQR)^†^	85 (61–122)	80 (56–124)	0.25
Early arrival (≤4.5 h); *n* (%)	221 (71%)	213 (77%)	0.10
Late arrival (>4.5 h); *n* (%)	92 (29%)	65 (23%)	
Known symptom onset; *n* (%)	186 (59%)	174 (63%)	0.43
Onset to door ≤4.5 h; *n* (%)	170 (91%)	165 (95%)	0.20
Onset to door >4.5 h; *n* (%)	16 (9%)	9 (5%)	
Unknown onset, LSN documented; *n* (%)	127 (41%)	104 (37%)	0.43
LSN-to-door ≤4.5 h; *n* (%)	51 (40%)	48 (46%)	0.36
LSN-to-door >4.5 h, *n* (%)	76 (60%)	56 (54%)	
Wake-up stroke; *n* (%)	69 (22%)	52 (19%)	0.32
Large vessel occlusion present; *n* (%)	98 (31%)	97 (35%)	0.36
Carotid artery; *n* (%)	17 (17%)	14 (14%)	0.14
Carotid T; *n* (%)	4 (4%)	11 (11%)	
M1; *n* (%)	33 (34%)	28 (29%)	
M2; *n* (%)	39 (40%)	31 (32%)	
Basilar artery; *n* (%)	2 (2%)	8 (8%)	
Vertebral artery; *n* (%)	3 (3%)	5 (5%)	
Endovascular thrombectomy; *n* (%)^‡^	71 (72%)	67 (69%)	0.60
IVT based on imaging modality; *n* (%)			
NCCT-based	197 (63%)	189 (68%)	0.20
CTP-based	114 (36%)	80 (29%)	0.048
MRI-based	2 (1%)	9 (3%)	0.02
TOAST classification; *n* (%)^#^			
Microangiopathy	47 (16%)	35 (13%)	0.36
Macroangiopathy	69 (23%)	71 (27%)	
Cardioembolic	101 (34%)	101 (38%)	
Unknown	76 (25%)	55 (21%)	
Other	8 (3%)	4 (2%)	

^*^ Defined as presence of coronary artery disease, peripheral artery disease or aortic plaque. ^†^ Exclusively patients with known time of symptom-onset (*n* = 360). ^‡^ Among patients with large vessel occlusion (LVO). ^#^ TOAST classification was assessed among patients with confirmed acute ischemic stroke (excluding stroke mimics; TNK, *n* = 301; rt-PA, *n* = 266).

AIS acute ischemic stroke; CTP, computed tomography perfusion; IVT, intravenous thrombolysis; IQR, interquartile range; LSN, last seen normal; mRS, modified Rankin Scale; TIA, transient ischemic attack; OAC, oral anticoagulation; NIHSS, National Institutes of Health Stroke Scale; NCCT, non-contrast computed tomography; MRI, magnetic resonance imaging.

### Stroke characteristics and imaging

Baseline stroke severity was similar between treatment groups, with comparable median NIHSS scores and distribution across severity categories (Table 1). Early presentation (≤4.5 h from symptom onset or last seen normal) occurred in 71% of TNK-treated and 77% of rt-PA–treated patients. The remaining patients presented more than 4.5 h after symptom onset or last seen normal, including both known-onset strokes presenting beyond 4.5 h and patients with unknown or unwitnessed onset, such as wake-up strokes. The distribution of presentation times is shown in Table 1, while the distribution of the actual IVT treatment time window is provided in Supplementary Table 1. IVT decision-making was primarily based on non-contrast CT, while multimodal imaging was used in selected cases, particularly in the extended time window. The prevalence of LVO and the distribution of occlusion sites were similar between treatment groups. Among patients with LVO, rates of subsequent EVT and successful reperfusion did not differ significantly. Stroke etiology according to TOAST classification was comparable between cohorts (*p* = 0.36), with cardioembolism representing the most frequent subtype in both groups.

### Procedural and early outcomes

Table 2 summarizes the primary workflow metrics and early in-hospital outcomes. Treatment with TNK was associated with significantly shorter workflow times. Median DTN time was 31 min (IQR 22–49) in the TNK group versus 35 min (IQR 26–54) in the rt-PA group (*p* = 0.004), primarily driven by shorter ITN intervals (13 vs. 16 min; *p* = 0.004). DTG time was numerically shorter in the TNK cohort but did not differ significantly between groups (70 vs. 76 min; *p* = 0.07), whereas DTI and DTR times did not differ significantly. IVT within 30 min of hospital arrival was more frequently achieved in TNK-treated patients (50% vs. 41%; *p* = 0.031). To evaluate temporal stability after implementation, DTN times were examined across semiannual intervals within the TNK period. Median DTN was 29 min (IQR 20–50) during June–November 2024 and 31 min (IQR 23–48) during December 2024–May 2025. The proportion of patients achieving DTN ≤ 30 min was 51 and 48%, respectively (Supplementary Table 2). The workflow advantage of TNK remained evident when restricting the analysis to patients treated within 4.5 h (DTN, 27 vs. 30 min, *p* = 0.015; ITN, 10 vs. 13 min, *p* = 0.015; DTG, 68 vs. 75 min, *p* = 0.043). In patients with evident stroke (NIHSS >4) treated within 4.5 h, DTN times remained significantly shorter with TNK (25 vs. 30 min, *p* = 0.007), whereas DTG times did not differ significantly between groups (67 vs. 74 min, *p* = 0.08; Table 2). Early neurological outcomes within the first 72 h were comparable between treatment groups. Major neurological improvement (≥ 4 NIHSS points) occurred in 40% of TNK-treated and 38% of rt-PA-treated patients (*p* = 0.59), while early neurological deterioration was observed in 7 and 12%, respectively (*p* = 0.11). Median NIHSS values during hospitalization and at discharge did not differ significantly (Figure 2). Among patients undergoing EVT, early recanalization prior to EVT occurred in 6% of TNK-treated and 1% of rt-PA-treated patients (*p* = 0.19). Rates of successful reperfusion following EVT (mTICI 2b–3) were similar between groups (93% vs. 91%; *p* = 0.68; see Table 2).

**Table 2:** In-hospital workflow times and early outcomes.

	TNK (*n* = 313)	rt-PA (*n* = 278)	*p* value
Procedure times, min; median (IQR)			
Door-to-needle (TNK *n* = 313; rt-PA *n* = 278)	31 (22–49)	35 (26–54)	0.004
Door-to-imaging (TNK *n* = 310; rt-PA *n* = 274)	17 (12–23)	18 (14–23)	0.11
Imaging-to-needle (TNK *n* = 311; rt-PA *n* = 275)	13 (7–25)	16 (9–29)	0.004
Door-to-groin (TNK *n* = 70; rt-PA *n* = 67)	70 (60–83)	76 (66–101)	0.07
Door-to-recanalization (TNK *n* = 64; rt-PA *n* = 61)	109 (91–131)	120 (95–155)	0.16
Door-to-needle within 30 min; *n* (%)	155 (50%)	113 (41%)	0.031
Procedure times for IVT ≤4.5 h (*n* = 407); median (IQR)			
Door-to-needle (TNK *n* = 210; rt-PA *n* = 197)	27 (20–44)	30 (24–43)	0.015
Door-to-imaging (TNK *n* = 208; rt-PA *n* = 195)	17 (11–22)	17 (13–21)	0.22
Imaging-to-needle (TNK *n* = 208; rt-PA *n* = 195)	10 (5–24)	13 (8–23)	0.015
Door-to-groin (TNK *n* = 48; rt-PA *n* = 44)	68 (58–81)	75 (66–99)	0.043
Door-to-recanalization (TNK *n* = 44; rt-PA *n* = 40)	104 (82–129)	120 (94–165)	0.05
Door-to-needle within 30 min; *n* (%)	123 (59%)	99 (50%)	0.09
Procedure times for evident strokes (NIHSS>4, time window ≤4.5 h, *n* = 261), min; median (IQR)			
Door-to-needle (TNK *n* = 132; rt-PA *n* = 129)	25 (19–43)	30 (23–42)	0.007
Door-to-imaging (TNK *n* = 130; rt-PA *n* = 128)	16 (11–19)	16 (13–20)	0.08
Imaging-to-needle (TNK *n* = 130; rt-PA *n* = 128)	10 (5–25)	13 (8–23)	0.027
Door-to-groin (TNK *n* = 47; rt-PA *n* = 37)	67 (58–80)	74 (66–85)	0.08
Door-to-recanalization (TNK *n* = 44; rt-PA *n* = 34)	104 (82–129)	114 (93–152)	0.17
Door-to-needle within 30 min; *n* (%)	86 (65%)	69 (53%)	0.06
Clinical in-hospital outcomes during the first 72 h			
Early major NIHSS improvement (≥4 within 72 h); *n* (%)	125 (40%)	105 (38%)	0.59
Early major NIHSS deterioration (≥4 by day 1 or 3)^*^; *n* (%)	18 (7%)	25 (12%)	0.11
Early recanalization pre-EVT^†^; *n* (%)	4 (6%)	1 (1%)	0.19
Radiological outcomes			
mTICI after EVT^†^; *n* (%)			0.99
mTICI 3	48 (68%)	45 (67%)	
mTICI 2c	10 (14%)	8 (12%)	
mTICI 2b	8 (11%)	8 (12%)	
mTICI 2a	1 (1%)	1 (1%)	
mTICI 0	4 (6%)	5 (7%)	
Successful reperfusion (mTICI 2b-3); *n* (%)	66 (93%)	61 (91%)	0.68

^*^Was assessed among patients who did not undergo EVT, as intubation for EVT may interfere with NIHSS assessment (TNK, *n* = 242; rt-PA, *n* = 211). ^†^ Among patients undergoing endovascular treatment (TNK, *n* = 71; rt-PA, *n* = 67). For door-to-groin and door-to-recanalization times, *n* indicates the number of patients with available measurements; missing values were due to incomplete documentation.

IQR, interquartile range; NIHSS, National Institutes of Health Stroke Scale; EVT, endovascular treatment; IVT, intravenous thrombolysis; mTICI, modified thrombolysis in cerebral infarction.

**Figure 2:**
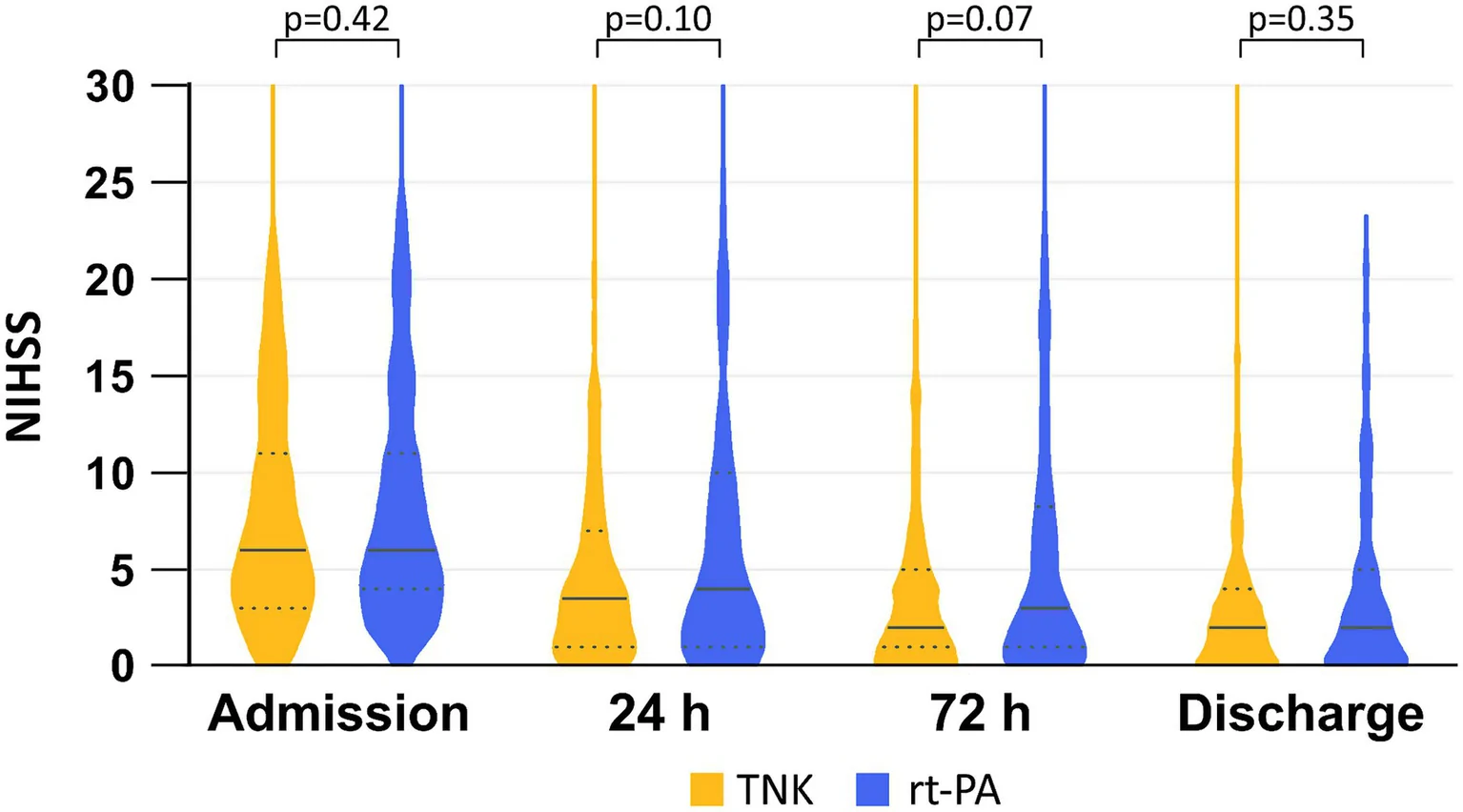
NIHSS trajectory over time. Violin plots showing the distribution of NIHSS scores, with median (solid line) and interquartile range (25th–75th percentiles; dotted lines), at admission, 24 h, 72 h, and discharge for TNK and rt-PA cohorts. The numbers of patients with available NIHSS assessments were 313 and 278 at admission, 310 and 274 at 24 h, 300 and 266 at 72 h, and 292 and 255 at discharge for the TNK and rt-PA cohorts, respectively. National Institutes of Health Stroke Scale.

### Safety outcomes overall

Any ICH occurred in 8% of TNK-treated and 7% of rt-PA–treated patients (unadjusted risk difference [RD] 1.1%, 95% CI –3.2 to 5.4%). Detailed unadjusted safety outcomes are provided in Supplementary Table 3. After multivariable adjustment, no statistically significant difference was observed between treatment groups (adjusted OR 1.30, 95% CI 0.69–2.42; adjusted RD 1.75%, 95% CI − 2.47 to 5.97%; Table 3). Symptomatic ICH rates were low across all definitions (SITS-MOST, ECASS-II, NINDS) and did not differ significantly between groups. PH type 2 and post-thrombolytic angioedema were rare and occurred at comparable frequencies (Table 3). Adjusted predicted probabilities for any ICH and excellent functional outcome across baseline NIHSS and age are shown in Supplementary Figure 1.

**Table 3:** Safety outcomes after multivariable adjustment comparing TNK and rt-PA.

Safety outcomes	TNK (*n* = 313)	rt-PA (*n* = 278)	Adjusted risk difference, % (95% CI)	Adjusted odds ratio (95% CI)	*p* value
Overall					
Any ICH; *n* (%)	26 (8%)	20 (7%)	+1.75 (−2.47 to 5.97)	1.30 (0.69 to 2.42)	0.42
sICH (SITS-MOST); *n* (%)	8 (3%)	4 (1%)	+1.51 (−0.8 to 3.81)	2.29 (0.62 to 8.38)	0.21
sICH (ECASS-II); *n* (%)	9 (3%)	8 (3%)	+0.47 (−2.20 to 3.14)	1.20 (0.43 to 3.31)	0.73
sICH (NINDS); *n* (%)	11 (4%)	10 (4%)	+0.25 (−2.7 to 3.24)	1.08 (0.44 to 2.64)	0.87
Parenchymal hematoma (PH2); *n* (%)	5 (2%)	2 (1%)	+1.63 (−0.96 to 4.23)	2.92 (0.50 to 17.04)	0.23
Angioedema after IVT; *n* (%)	7 (2%)	4 (1%)	+1.18 (−1.34 to 3.70)	1.77 (0.51 to 6.13)	0.37
Subgroups					
IVT Time window ≤ 4.5 h (TNK *n* = 210; rt-PA *n* = 197)					
Any ICH in IVT within 4.5 h; *n* (%)	20 (10%)	15 (8%)	+2.33 (−3.00 to +7.66)	1.37 (0.66 to 2.84)	0.40
sICH in IVT within 4.5 h (SITS-MOST); *n* (%)	7 (3%)	4 (2%)	+1.66 (−1.57 to 4.90)	1.97 (0.52 to 7.55)	0.32
sICH in IVT within 4.5 h (ECASS-II); *n* (%)	8 (4%)	8 (4%)	+0.23 (−3.54 to 4.01)	1.07 (0.38 to 3.04)	0.90
sICH in IVT within 4.5 h (NINDS); *n* (%)	8 (4%)	8 (4%)	+0.11 (−3.68 to 3.90)	1.03 (0.36 to 2.92)	0.96
IVT Time window > 4.5 h (TNK *n* = 103; rt-PA *n* = 81)					
Any ICH in IVT > 4.5 h; *n* (%)	6 (6%)	5 (6%)	+2.04 (−4.79 to 8.88)	1.48 (0.40 to 5.50)	0.56
sICH in IVT > 4.5 h (SITS-MOST); *n* (%)	1 (1%)	0 (0%)	NE	NE	-
sICH in IVT > 4.5 h (ECASS-II); *n* (%)	1 (1%)	0 (0%)	NE	NE	-
sICH in IVT > 4.5 h (NINDS); *n* (%)	3 (3%)	2 (2%)	+1.73 (−3.20 to 6.66)	1.98 (0.29 to 13.62)	0.49
Patients under concurrent OAC (TNK *n* = 43; rt-PA *n* = 42)					
Any ICH; *n* (%)	4 (9%)	4 (10%)	+1.65 (−10.98 to 14.27)	1.24 (0.24 to 6.46)	0.80
sICH (SITS-MOST); *n* (%)	2 (5%)	2 (5%)	+1.90 (−7.88 to 11.68)	1.62 (0.14 to 19.19)	0.70
sICH (ECASS-II); *n* (%)	2 (5%)	3 (7%)	−2.45 (−13.23 to 8.32)	0.62 (0.07 to 5.23)	0.66
sICH (NINDS); *n* (%)	2 (5%)	3 (7%)	−2.45 (−13.23 to 8.32)	0.62 (0.07 to 5.23)	0.66
Patients with LVO (TNK *n* = 98; rt-PA *n* = 97)					
Any ICH in LVO; *n* (%)	10 (10%)	15 (15%)	−5.46 (−14.72 to 3.81)	0.59 (0.24 to 1.45)	0.25
sICH (SITS-MOST); *n* (%)	2 (2%)	2 (2%)	+1.82 (−2.28 to 5.93)	3.22 (0.21 to 49.95)	0.40
sICH (ECASS-II); *n* (%)	3 (3%)	6 (6%)	−2.02 (−7.87 to 3.84)	0.60 (0.13 to 2.74)	0.51
sICH (NINDS); *n* (%)	2 (2%)	7 (7%)	−4.43 (−10.22 to 1.36)	0.31 (0.06 to 1.60)	0.16
Patients with minor stroke (NIHSS 1–4; TNK *n* = 101; rt-PA *n* = 92)					
Any ICH in minor stroke; *n* (%)	4 (4%)	1 (1%)	+3.49 (−2.08 to 9.06)	3.51 (0.38 to 32.58)	0.27
sICH (SITS-MOST); *n* (%)	1 (1%)	0 (0%)	NE	NE	-
sICH (ECASS-II); *n* (%)	1 (1%)	0 (0%)	NE	NE	-
sICH (NINDS); *n* (%)	3 (3%)	0 (0%)	NE	NE	-
Patients with moderate stroke (NIHSS 5–15; TNK *n* = 161; rt-PA *n* = 143)					
Any ICH in moderate stroke; *n* (%)	16 (10%)	10 (7%)	+3.46 (−2.73 to 9.65)	1.60 (0.68 to 3.72)	0.28
sICH (SITS-MOST); *n* (%)	5 (3%)	2 (1%)	+3.61 (−2.92 to 10.15)	2.45 (0.45 to 13.35)	0.30
sICH (ECASS-II); *n* (%)	6 (4%)	3 (2%)	+2.66 (−2.94 to 8.25)	1.94 (0.46 to 8.08)	0.37
sICH (NINDS); *n* (%)	6 (4%)	4 (3%)	+1.19 (−2.82 to 5.20)	1.46 (0.40 to 5.36)	0.57
Patients with severe stroke (NIHSS ≥16; TNK *n* = 38; rt-PA *n* = 41)					
Any ICH in severe stroke; *n* (%)	6 (16%)	9 (22%)	−9.57 (−27.22 to 8.08)	0.50 (0.13 to 1.84)	0.29
sICH (SITS-MOST); *n* (%)	2 (5%)	2 (5%)	NE	NE	-
sICH (ECASS-II); *n* (%)	2 (5%)	5 (12%)	NE	NE	-
sICH (NINDS); *n* (%)	2 (5%)	6 (15%)	NE	NE	-
Patients aged >80 years (TNK *n* = 112; rt-PA *n* = 107)					
Any ICH in patients aged >80 years; *n* (%)	14 (13%)	7 (7%)	+6.52 (−1.06 to 14.10)	2.30 (0.84 to 6.27)	0.10
sICH (SITS-MOST); *n* (%)	3 (3%)	2 (2%)	NE	NE	-
sICH (ECASS-II); *n* (%)	3 (3%)	3 (3%)	+0.14 (−4.07 to 4.36)	1.06 (0.18 to 6.18)	0.95
sICH (NINDS); *n* (%)	5 (4%)	4 (4%)	+1.24 (−3.99 to 6.47)	1.41 (0.33 to 6.07)	0.65

Adjusted odds ratios and risk differences were derived from multivariable logistic regression models adjusted for age, baseline NIHSS, treatment within 4.5 h, endovascular thrombectomy, OAC, dual antiplatelet therapy, and LVO.

CI, confidence interval; ICH, intracranial hemorrhage; IVT, intravenous thrombolysis; LVO, large vessel occlusion; NE, not estimable because of sparse outcome events and/or complete or quasi-complete separation in the multivariable model; NIHSS, National Institutes of Health Stroke Scale; OAC, oral anticoagulation; PH2, parenchymal hematoma type 2; sICH, symptomatic intracranial hemorrhage.

### Subgroup and propensity score analyses

In subgroup analyses stratified by treatment time window, OAC, LVO, stroke severity and age > 80 years, adjusted models did not demonstrate statistically significant differences in any ICH between treatment groups (Table 3). Estimates for symptomatic ICH were also not statistically significant where estimable, although precision was limited in smaller subgroups with few or no events. Confidence intervals were wider in smaller strata. A forest plot of adjusted ORs for any ICH is presented in Supplementary Figure 2. Hemorrhagic complications stratified by treatment time window and OAC status are presented in Supplementary Table 4. Results remained consistent in propensity score–matched and AIPTW-weighted analyses (Supplementary Table 5).

### Propensity score-matched analysis

After propensity score matching, 255 TNK-treated and 255 rt-PA-treated patients were available for analysis. Covariate balance was substantially improved after matching, with SMD below 0.1 for all matching covariates (Supplementary Table 6; Supplementary Figure 3). In the propensity score-matched cohort, TNK remained associated with shorter DTN times (31 vs. 36 min; *p* = 0.005), ITN intervals (12 vs. 16 min; *p* = 0.020), and DTG times (70 vs. 75 min; *p* = 0.031). DTN within 30 min was more frequently achieved with TNK (49% vs. 40%; *p* = 0.032). Other workflow parameters did not differ significantly (Supplementary Table 7). Median mRS at discharge was 3 (IQR 2–3) in the TNK group as well as 3 (IQR 2–4) in the rt-PA group (Figure 3). Rates of excellent (mRS 0–1) and favorable functional outcome (mRS 0–2), both in absolute terms and relative to premorbid functional status, did not differ significantly between groups (Supplementary Table 8). In-hospital mortality was not statistically different between treatment groups. Length of hospital stay, discharge destination and the proportion of stroke mimics were comparable between groups. Adjusted predicted probabilities for functional outcome across baseline NIHSS and age are illustrated in Supplementary Figure 1.

**Figure 3:**
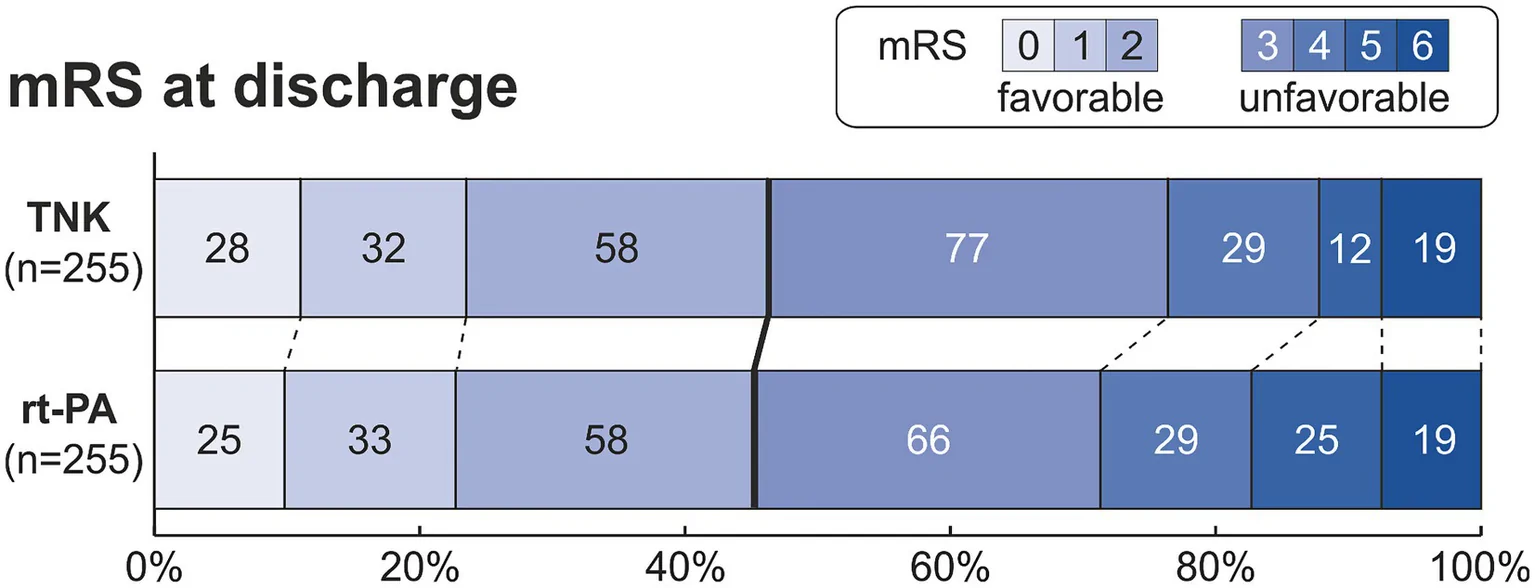
mRS distribution at discharge after propensity score matching. Stacked bar plot showing mRS scores at discharge for TNK versus rt-PA groups after propensity score matching. mRS, modified Rankin Scale; PSM, propensity score matching.

## Discussion

In this real-world cohort of consecutive IVT-treated patients, institutional implementation of TNK was associated with shorter door-to-needle and imaging-to-needle times without evidence of an increased risk of hemorrhagic complications. Although the absolute reduction in DTN time was modest (31 vs. 35 min), even small decreases in treatment delay are clinically meaningful at a population level, given the time-dependent nature of neuronal loss in AIS. Importantly, no increase in hemorrhagic complications was observed in formal off-label scenarios, including treatment beyond 4.5 h and thrombolysis under OAC. These findings were consistent across multivariable-adjusted, propensity score–matched and weighted analyses, supporting the robustness of the observed safety profile despite baseline differences between treatment groups.

The shorter treatment initiation with TNK supports and extends findings from our prior institutional analysis and other real-world cohorts demonstrating that single-bolus administration facilitates streamlined workflows (16, 22–25). In our cohort, DTI times were unchanged, whereas ITN intervals were significantly shorter (13 vs. 16 min), indicating that the improvement in DTN time was primarily driven by simplified drug preparation and bolus administration rather than structural workflow modifications. This logistical advantage likely contributes not only to earlier IVT initiation but also to more efficient procedural preparation for EVT. In particular, the avoidance of a prolonged infusion and additional intravenous access may simplify management in anesthetized or intubated patients (26, 27). Consistent with this interpretation, DTG times were numerically shorter with TNK in the overall cohort (70 vs. 76 min), with statistically significant differences observed in the subgroup of patients treated within 4.5 h (68 vs. 75 min; *p* = 0.043) and in the propensity score-matched cohort (70 vs. 75 min; *p* = 0.031). Importantly, semiannual analyses demonstrated sustained DTN performance throughout the post-transition period, without evidence of temporal attenuation. This finding argues against a transient short-term training or implementation effect as the primary explanation for the observed workflow improvement and suggests stable integration of TNK into routine clinical practice.

Beyond workflow improvements, this study addresses clinical scenarios that continue to generate uncertainty in routine practice, particularly treatment beyond 4.5 h and thrombolysis under OAC. Notably, 31% of patients in our cohort received IVT beyond 4.5 h, representing a substantial real-world population relevant to the extended-window evidence base. In our cohort, TNK use in the extended time window was not associated with increased rates of any or symptomatic ICH compared with rt-PA. The use of imaging-selected IVT beyond 4.5 h is supported by prior evidence from randomized trials and individual-patient data meta-analyses and was incorporated into the 2021 ESO guidelines (28). More recently, randomized trials have specifically evaluated TNK between 4.5 and 24 h, including TRACE-III, TIMELESS and CHABLIS-T II, which – despite heterogeneous functional outcomes – did not demonstrate a consistent excess in hemorrhagic risk when patient selection was guided by advanced imaging (7–9). Although individual studies have reported numerically higher symptomatic hemorrhage rates, differences in study populations and comparators limit direct cross-trial comparisons. Interpretation of outcomes in the extended window requires caution because differences in patient selection and concomitant EVT may contribute to heterogeneity in treatment effects across studies (29). In our cohort, patients treated beyond 4.5 h were selected based on CT perfusion findings, which may limit extrapolation to unselected late-window populations. Overall, current evidence does not indicate a clear increase in hemorrhagic risk with TNK in imaging-selected late-window patients (30).

Among patients receiving OAC who fulfilled predefined laboratory criteria for IVT eligibility, TNK was not associated with increased rates of any or symptomatic ICH compared with rt-PA. This finding is consistent with emerging registry data supporting the safety of carefully selected anticoagulated patients undergoing thrombolysis (31–33). Even in the combined high-risk constellation of extended time window and concurrent anticoagulation, no excess hemorrhagic signal was observed, although event numbers were limited. Similarly, no increase in ICH was detected in patients aged >80 years. These findings should be interpreted cautiously in light of recent data reporting less favorable 90-day functional outcomes with TNK in very elderly patients with minor stroke (13, 34). Taken together, these exploratory analyses do not suggest that thrombolytic agent selection confers additional hemorrhagic risk in these higher-risk subgroups.

Early recanalization prior to EVT occurred numerically more often in TNK-treated patients, although overall rates were low and differences did not reach statistical significance. In a comprehensive stroke center operating under a direct-admission (“mothership”) paradigm, rapid progression from IVT to thrombectomy results in minimal dwell time between thrombolysis and EVT, thereby limiting the opportunity for pharmacological recanalization to occur. In contrast, transfer-based models may provide longer intervals during which thrombolytic therapy can exert its effect (35).

Beyond workflow efficiency and hemorrhagic safety, our secondary clinical outcomes provide additional information on the real-world implementation of TNK in a heterogeneous AIS population. No statistically significant differences were observed in early major neurological improvement, early neurological deterioration, or successful reperfusion following EVT. Similarly, after propensity score matching, no statistically significant differences were observed in dichotomized functional outcomes at discharge or in-hospital mortality. These findings are broadly consistent with the available evidence showing similar clinical outcomes with TNK and rt-PA and suggest that TNK implementation was not associated with an apparent disadvantage in early neurological or in-hospital functional outcomes (3, 4). In this implementation setting, the principal measurable advantage of TNK appears to be improved workflow efficiency rather than demonstrable superiority in clinical recovery.

Several limitations merit consideration. The study’s before–after design precludes causal inference and leaves the possibility of residual confounding or secular trends. Although no structural workflow modifications were implemented apart from the pharmacological transition, unmeasured temporal influences cannot be fully excluded. Second, the single-center setting may limit generalizability, particularly to lower-volume hospitals. Third, subgroup analyses, especially in patients treated beyond 4.5 h under OAC, were constrained by small event numbers. Finally, functional outcome was assessed at discharge rather than at 90 days. Discharge mRS represents an early indicator of functional status and cannot fully capture long-term recovery or return to pre-stroke activities. Consequently, these results should not be directly compared with 90-day outcomes reported in randomized trials and should be interpreted cautiously.

## Conclusion

In a high-volume comprehensive stroke center, institutional transition to TNK was associated with shorter treatment initiation without evidence of increased in-hospital hemorrhagic risk, including in extended time windows and carefully selected anticoagulated patients. The primary measurable advantage of TNK in this implementation setting appears to be operational efficiency rather than demonstrable clinical superiority.

## Data Availability

The datasets presented in this article are not readily available because the dataset contains clinical patient data and cannot be made publicly available due to local data protection regulations and patient privacy restrictions. Access to anonymized data may be considered upon reasonable request to the corresponding author and with permission of the local ethics committee. Requests to access the datasets should be directed to AS, alexander.sekita@uk-erlangen.de.

## Glossary

AIS: Acute ischemic stroke
AIPTW: Augmented inverse probability of treatment weighting
CI: Confidence interval
CT: Computed tomography
DTG: Door-to-groin
DTI: Door-to-imaging
DTN: Door-to-needle
DTR: Door-to-recanalization
ECASS II: European Cooperative Acute Stroke Study II
EVT: Endovascular treatment
ICH: Intracranial hemorrhage
INR: International normalized ratio
IQR: Interquartile range
ITN: Imaging-to-needle
IVT: Intravenous thrombolysis
LVO: Large vessel occlusion
MRI: Magnetic resonance imaging
mRS: Modified Rankin Scale
mTICI: Modified Thrombolysis in Cerebral Infarction
NIHSS: National Institutes of Health Stroke Scale
NINDS: National Institute of Neurological Disorders and Stroke
OAC: Oral anticoagulation
PH: Parenchymal hematoma
SMD: Standardized mean difference
RD: Risk difference
rt-PA: Recombinant tissue plasminogen activator or alteplase
SITS-MOST: Safe Implementation of Thrombolysis in Stroke – Monitoring Study
SOP: Standard operating procedure
TNK: tenecteplase
