# Supplementary material for: Implementation of tenecteplase for acute ischemic stroke: workflow and safety in routine care including off-label use
Source: Front Neurol. 2026 Oct 28;17:1882561. doi: 10.3389/fneur.2026.1882561 (PMC13751450; doi:10.3389/fneur.2026.1882561)
Supplement: Supplementary file 1 [file Data_Sheet_1.PDF]

Supplementary Material

Sekita et al. Implementation of Tenecteplase for Acute Ischemic Stroke: Workflow and Safety in Routine Care Including Off-Label Use

Content:

Supplementary Methods

sTable 1: **IVT time window distribution**

sTable 2: **Door-to-needle times across semiannual intervals after institutional transition to Tenecteplase**

sTable 3: **Unadjusted Safety Outcomes Comparing tenecteplase and alteplase**

sTable 4: **Hemorrhagic complications by time window and OAC status**

sTable 5: **Unadjusted and propensity score-based analyses of intracranial hemorrhage**

sTable 6: **Baseline characteristics and covariate balance before and after matching**

sTable 7: **Primary workflow outcomes after propensity score matching**

sTable 8: **Clinical outcomes after propensity score matching**

sFigure 1: **Adjusted predicted probabilities by treatment, stroke severity, and age**

sFigure 2: **Adjusted odds ratios for any intracranial hemorrhage by subgroup**

sFigure 3: **Covariate balance before and after propensity score matching.**

Supplementary Methods:

Implementation of Tenecteplase and Institutional Transition Process

The transition from alteplase (rt-PA) to tenecteplase (TNK) as the standard thrombolytic agent at our tertiary comprehensive stroke center was conducted in a structured and stepwise manner. Prior to implementation, institutional standard operating procedures (SOPs) for acute stroke management were revised to incorporate TNK administration, dosing, and eligibility criteria in accordance with contemporary guideline recommendations.

In addition to routinely performed simulation-based team training for intravenous thrombolysis in the emergency department, all physicians and nursing staff involved in acute stroke care participated in dedicated training sessions beginning in April 2024. These sessions focused on pharmacological differences between rt-PA and TNK, preparation and administration procedures, dosing safety, and workflow integration. Particular emphasis was placed on avoiding dosing errors by exclusively using stroke-specific TNK vials (maximum dose 25 mg) and adapting emergency room workflows to single-bolus administration.

The institutional transition to TNK as the default thrombolytic agent was completed in June 2024. The implementation phase and early institutional experience during the first six months after transition have been reported previously. The present study incorporates this initial cohort but represents a complete and independent analysis including an extended observation period and additional statistical analyses not performed in the initial report.

Analysis populations and descriptive comparisons

The full cohort comprised 591 IVT-treated patients: 313 receiving tenecteplase (TNK) and 278 receiving alteplase (rt-PA). Analyses distinguished comparisons in the full unmatched cohort, comparisons within the propensity score-matched cohort, and augmented inverse probability of treatment weighting analyses in the full cohort.

Continuous variables were summarized as medians with interquartile ranges. Categorical variables were reported as counts and percentages using the relevant analysis population as the denominator. For outcomes with incomplete measurements or restricted eligibility, available sample sizes were reported separately. Unmatched continuous outcomes were compared using the Mann–Whitney U test; categorical outcomes were compared using Pearson's chi-square or Fisher's exact test. The unadjusted comparisons in Supplementary Table 5 used Pearson's chi-square test without continuity correction.

Unadjusted and multivariable regression analyses

Unadjusted logistic regression models included treatment group as the sole explanatory variable. Multivariable logistic regression models were fitted separately for hemorrhagic outcomes, with TNK compared against rt-PA as the reference treatment. The adjustment set included age, baseline NIHSS, treatment within 4.5 hours, endovascular thrombectomy, oral anticoagulation, dual antiplatelet therapy, and large-vessel occlusion. Odds ratios and 95% confidence intervals were obtained from the treatment coefficients and their standard errors.

Adjusted risks were estimated by marginal standardization: predicted event probabilities were calculated under TNK and rt-PA for the covariate profiles of the relevant analysis population and averaged within each treatment scenario. The adjusted risk difference was the difference between these standardized risks, defined as TNK minus rt-PA. Table 3 presents adjusted risk differences in percentage points and adjusted odds ratios; Supplementary Table 3 presents the corresponding unadjusted estimates.

P values for treatment effects were based on Wald chi-square tests of the treatment coefficients in the logistic regression models.

Subgroup analyses

Subgroup analyses were conducted according to treatment time window (≤4.5 versus >4.5 hours), concurrent oral anticoagulation, large-vessel occlusion, admission NIHSS category (1–4, 5–15, or ≥16), and age >80 years. Models were fitted within the respective subgroups. A binary covariate defining a subgroup was omitted when it was constant within that subgroup.

Adjusted predicted probabilities in Supplementary Figure 1 were derived from multivariable logistic regression models. The model for any intracranial hemorrhage included treatment group, baseline NIHSS, age, large-vessel occlusion, endovascular thrombectomy, premorbid mRS, treatment within 4.5 hours, and dual antiplatelet therapy. The model for excellent functional outcome at discharge (mRS 0–1) included the same covariates. Predicted probabilities were plotted according to baseline NIHSS and age, respectively.

Propensity score estimation and matching

The propensity score was defined as the probability of receiving TNK conditional on the selected covariates. It was estimated by logistic regression including age and CHA_2DS_2-VASc score as continuous variables and hypertension and treatment within 4.5 hours as binary variables.

One-to-one nearest-neighbor matching without replacement was performed on the logit of the propensity score. The caliper was 0.2 times the standard deviation of the logit propensity scores in the full cohort, corresponding to 0.06051 logit units. Ascending propensity-score order was selected during reanalysis after comparison of alternative matching orders. Matching retained 255 patients per treatment group, forming 255 matched pairs.

Covariate balance was evaluated using absolute standardized mean differences (SMDs). For continuous variables, the denominator was the square root of the average of the two full-cohort group sample variances. For binary variables, the denominator was the square root of the average of $p(1-p)$ in the two full-cohort treatment groups. These pre-matching denominators were retained when calculating post-matching SMDs. An absolute SMD below 0.1 indicated acceptable balance. Balance is reported in Supplementary Table 6 and displayed for the four propensity-score covariates in Supplementary Figure 3.

Outcome comparisons after matching

Binary outcomes were compared using the exact McNemar test, which evaluates discordant outcomes within matched pairs. This procedure was used for hemorrhagic outcomes in Supplementary Table 5, DTN ≤30 minutes in Supplementary Table 7, and dichotomous clinical outcomes in Supplementary Table 8.

Continuous outcomes and the comparison accompanying the median discharge mRS were analyzed using paired Wilcoxon signed-rank tests. Tests were restricted to pairs with measurements available for both patients. For workflow outcomes in Supplementary Table 7, the normal approximation with tie and continuity corrections was used.

Descriptive medians and interquartile ranges summarize all available measurements within each treatment group. The number of available measurements therefore need not equal the number of complete pairs contributing to a paired test. Supplementary Table 7 reports these quantities separately. In particular, the paired comparisons of door-to-groin and door-to-recanalization times included 12 and 10 complete pairs, respectively. Differences between the displayed group medians should not be interpreted as estimates of within-pair differences.

The complete discharge mRS distribution and discharge destination were compared using Stuart–Maxwell tests of marginal homogeneity for paired categorical data.

Risk differences in the matched cohort

For any intracranial hemorrhage and sICH according to SITS-MOST, marginal risk differences were obtained from logistic regression containing treatment group as the sole explanatory variable. Pair-clustered robust variance was used to calculate Wald confidence intervals and p values.

Because these models contained no additional covariates, the estimated risks equaled the observed event proportions in the matched treatment groups. The risk-difference point estimate therefore equaled the crude difference between these proportions. Pair clustering affected variance estimation and inference, rather than the point estimate.

Supplementary Table 5 separately reports exact McNemar p values for paired binary comparisons and Wald p values accompanying the risk-difference estimates. These p values may differ because they arise from distinct inferential procedures.

Augmented inverse probability of treatment weighting

Augmented inverse probability of treatment weighting (AIPTW), a doubly robust approach combining treatment and outcome models, was performed in the full cohort for any intracranial hemorrhage and symptomatic intracranial hemorrhage according to SITS-MOST. The target estimand was the average treatment effect on the treated (ATT), defined as the average difference in event probability under TNK versus rt-PA among patients who received TNK.

The treatment model estimated the propensity to receive TNK using logistic regression including age, hypertension, CHA_2DS_2-VASc score, and treatment within 4.5 hours. Separate logistic outcome models for the two hemorrhagic endpoints were fitted among rt-PA recipients and included prior antiplatelet therapy, admission NIHSS, age, and endovascular thrombectomy. These models predicted the counterfactual event risk under rt-PA for TNK recipients.

The normalized ATT estimator combined outcome-model predictions of the counterfactual event risk under rt-PA for TNK recipients with propensity-odds weighting of rt-PA recipients. Treatment effects were expressed as absolute risk differences, calculated as TNK minus rt-PA. Sandwich standard errors accounted for estimation of both the propensity-score and outcome models, and Wald 95% confidence intervals and p values were reported. No interaction terms were included.

AIPTW targeted the entire TNK cohort, whereas the matching analysis described the TNK patients retained after matching and their matched controls. The estimates may therefore differ because both the analysis populations and estimation procedures differ.

Effect scales and reporting

Risk differences were defined as TNK minus rt-PA. Positive values indicate a higher event risk under TNK. In Supplementary Table 5, risk differences are expressed on the probability scale; multiplication by 100 converts them to percentage points. Odds ratios for hemorrhagic outcomes above 1 indicate higher odds under TNK.

All tests were two-sided. The statistical test used for each post-matching comparison is identified in the corresponding table footnote.

Supplementary Table 1: IVT time window distribution.

	**TNK** (n=313)	**rt-PA** (n=278)	***P* Value**
IVT time window ; n (%)			
≤1.5 h	53 (17%)	49 (18%)	0.82
1.5 - 3 h	112 (36%)	106 (38%)	0.56
3 - 4.5 h	45 (14%)	42 (15%)	0.80
4.5 - 6 h	24 (8%)	21 (8%)	0.96
6 - 7.5 h	17 (5%)	9 (3%)	0.19
7.5 - 9 h	15 (5%)	9 (3%)	0.34
9 - 12 h	19 (6%)	16 (6%)	0.87
12 - 18 h	22 (7%)	23 (8%)	0.57
18 - 24 h	6 (2%)	3 (1%)	0.41

Abbreviations: IVT, intravenous thrombolysis.

Supplementary Table 2: Door-to-needle times across semiannual intervals after institutional transition to Tenecteplase

TNK period	n	DTN median (IQR), min	ITN median (IQR), min	DTN ≤30 min, n (%)
June 2024–November 2024	138	29 (20–50)	14 (9–27)	71 (51%)
December 2024–May 2025	175	31 (23–48)	11 (5–24)	84 (48%)

Abbreviations: DTN, door-to-needle; ITN, imaging-to-needle; IQR, interquartile range.

Supplementary Table 3: Unadjusted Safety Outcomes Comparing Tenecteplase and Alteplase

**Safety outcomes**	**TNK** (n=313)	**rt-PA** (n=278)	**Unadjusted risk difference, % (95% CI)**	**Unadjusted odds ratio (95% CI)**	***P* Value**
**Overall**					
Any ICH; n (%)	26 (8%)	20 (7%)	1.11 (-3.20 to 5.42)	1.17 (0.64 to 2.14)	0.61
sICH (SITS-MOST); n (%)	8 (3%)	4 (1%)	1.12 (-1.12 to 3.36)	1.80 (0.54 to 6.03)	0.34
sICH (ECASS-II); n (%)	9 (3%)	8 (3%)	0.00 (-2.70 to 2.70)	1.00 (0.38 to 2.63)	0.99
sICH (NINDS); n (%)	11 (4%)	10 (4%)	-0.08 (-3.08 to 2.91)	0.98 (0.41 to 2.33)	0.96
Parenchymal hematoma (PH2); n (%)	5 (2%)	2 (1%)	0.88 (-0.83 to 2.59)	2.24 (0.43 to 11.64)	0.34
Angioedema after IVT; n (%)	7 (2%)	4 (1%)	0.80 (-1.36 to 2.95)	1.57 (0.45 to 5.41)	0.48
**Subgroups**					
- Time window ≤ 4.5h (TNK n=210; rt-PA n=197)					
Any ICH in IVT within 4.5h; n (%)	20 (10%)	15 (8%)	1.91 (-3.52 to 7.34)	1.28 (0.63 to 2.57)	0.49
sICH in IVT within 4.5h (SITS-MOST); n (%)	7 (3%)	4 (2%)	1.30 (-1.82 to 4.43)	1.66 (0.48 to 5.77)	0.42
sICH in IVT within 4.5h (ECASS-II); n (%)	8 (4%)	8 (4%)	-0.25 (-4.03 to 3.53)	0.94 (0.34 to 2.54)	0.90
sICH in IVT within 4.5h (NINDS); n (%)	8 (4%)	8 (4%)	-0.25 (-4.03 to 3.53)	0.94 (0.34 to 2.54)	0.90
- Time window > 4.5h (TNK n=103; rt-PA n=81)					
Any ICH in IVT > 4.5h; n (%)	6 (6%)	5 (6%)	-0.35 (-7.27 to 6.58)	0.94 (0.28 to 3.20)	0.92
sICH in IVT >4.5h (SITS-MOST); n (%)	1 (1%)	0 (0%)	0.97 (-0.92 to 2.86)	NE	-
sICH in IVT >4.5h (ECASS-II); n (%)	1 (1%)	0 (0%)	0.97 (-0.92 to 2.86)	NE	-
sICH in IVT >4.5h (NINDS); n (%)	3 (3%)	2 (2%)	0.44 (-4.24 to 5.13)	1.18 (0.19 to 7.27)	0.85
- Patients under concurrent OAC (TNK n=43; rt-PA n=42)					
Any ICH; n (%)	4 (9%)	4 (10%)	-0.22 (-12.64 to 12.20)	0.97 (0.23 to 4.18)	0.97
sICH (SITS-MOST); n (%)	2 (5%)	2 (5%)	-0.11 (-9.12 to 8.89)	0.98 (0.13 to 7.27)	0.98
sICH (ECASS-II); n (%)	2 (5%)	3 (7%)	-2.49 (-12.51 to 7.52)	0.63 (0.10 to 4.00)	0.63
sICH (NINDS); n (%)	2 (5%)	3 (7%)	-2.49 (-12.51 to 7.52)	0.63 (0.10 to 4.00)	0.63
- Patients with LVO (TNK n=98; rt-PA n=97)					
Any ICH in LVO; n (%)	10 (10%)	15 (15%)	-5.26 (-14.62 to 4.10)	0.62 (0.26 to 1.46)	0.27
sICH (SITS-MOST); n (%)	2 (2%)	2 (2%)	-0.02 (-4.00 to 3.96)	0.99 (0.14 to 7.17)	0.99
sICH (ECASS-II); n (%)	3 (3%)	6 (6%)	-3.12 (-9.01 to 2.76)	0.48 (0.12 to 1.97)	0.30
sICH (NINDS); n (%)	2 (2%)	7 (7%)	-5.18 (-11.04 to 0.69)	0.27 (0.05 to 1.32)	0.11
- Patients with minor stroke (NIHSS 1–4; TNK n=101; rt-PA n=92)					
Any ICH in minor stroke; n (%)	4 (4%)	1 (1%)	2.87 (-1.48 to 7.23)	3.75 (0.41 to 34.21)	0.24
sICH (SITS-MOST); n (%)	1 (1%)	0 (0%)	0.99 (-0.94 to 2.92)	NE	-
sICH (ECASS-II); n (%)	1 (1%)	0 (0%)	0.99 (-0.94 to 2.92)	NE	-
sICH (NINDS); n (%)	3 (3%)	0 (0%)	2.97 (-0.34 to 6.28)	NE	-
- Patients with moderate stroke (NIHSS 5–15; TNK n=161; rt-PA n=143)					
Any ICH in moderate stroke; n (%)	16 (10%)	10 (7%)	2.94 (-3.29 to 9.18)	1.47 (0.64 to 3.35)	0.36
sICH (SITS-MOST); n (%)	5 (3%)	2 (1%)	1.71 (-1.59 to 5.01)	2.26 (0.43 to 11.83)	0.34
sICH (ECASS-II); n (%)	6 (4%)	3 (2%)	1.63 (-2.12 to 5.38)	1.81 (0.44 to 7.36)	0.41
sICH (NINDS); n (%)	6 (4%)	4 (3%)	0.93 (-3.05 to 4.91)	1.35 (0.37 to 4.87)	0.65
- Patients with severe stroke (NIHSS ≥16; TNK n=38; rt-PA n=41)					
Any ICH in severe stroke; n (%)	6 (16%)	9 (22%)	-6.16 (-23.34 to 11.01)	0.67 (0.21 to 2.09)	0.49
sICH (SITS-MOST); n (%)	2 (5%)	2 (5%)	0.39 (-9.30 to 10.07)	1.08 (0.14 to 8.10)	0.94
sICH (ECASS-II); n (%)	2 (5%)	5 (12%)	-6.93 (-19.21 to 5.35)	0.40 (0.07 to 2.20)	0.29
sICH (NINDS); n (%)	2 (5%)	6 (15%)	-9.37 (-22.31 to 3.57)	0.32 (0.06 to 1.72)	0.19
- Patients aged >80 years (TNK n=112; rt-PA n=107)					
Any ICH in patients aged >80 years; n (%)	14 (13%)	7 (7%)	5.96 (-1.75 to 13.67)	2.04 (0.79 to 5.27)	0.14
sICH (SITS-MOST); n (%)	3 (3%)	2 (2%)	0.81 (-3.13 to 4.75)	1.44 (0.24 to 8.82)	0.69
sICH (ECASS-II); n (%)	3 (3%)	3 (3%)	-0.13 (-4.45 to 4.20)	0.95 (0.19 to 4.83)	0.96
sICH (NINDS); n (%)	5 (4%)	4 (4%)	0.73 (-4.52 to 5.97)	1.20 (0.31 to 4.61)	0.79

Abbreviations: CI, confidence interval; ICH, intracranial hemorrhage; IVT, intravenous thrombolysis; LVO, large vessel occlusion; NE, not estimable because of sparse outcome events and/or complete or quasi-complete separation in the regression model; NIHSS, National Institutes of Health Stroke Scale; OAC, oral anticoagulation; PH2, parenchymal hematoma type 2; sICH, symptomatic intracranial hemorrhage.

Supplementary Table 4: Hemorrhagic complications by time window and OAC status.

	IVT ≤ 4.5 h	IVT > 4.5h	∑
**Overall (n=591)**			
**Any ICH (n=46)**			
**No OAC**	354 (60%)<br>Any ICH: 29 (63%)	152 (26%)<br>Any ICH: 9 (20%)	506 (86%)<br>Any ICH: 38 (83%)
**OAC**	53 (9%)<br>Any ICH: 6 (13%)	32 (5%)<br>Any ICH: 2 (4%)	85 (14%)<br>Any ICH: 8 (17%)
∑	407 (69%)<br>Any ICH: 35 (76%)	184 (31%)<br>Any ICH: 11 (24%)	591 (100%)<br>Any ICH: 46 (100%)
**sICH (n=12)**			
**No OAC**	354 (60%)<br>sICH: 8 (67%)	152 (26%)<br>sICH: 0 (0%)	506 (86%)<br>sICH: 8 (67%)
**OAC**	53 (9%)<br>sICH: 3 (25%)	32 (5%)<br>sICH: 1 (8%)	85 (14%)<br>sICH: 4 (33%)
∑	407 (69%)<br>sICH: 11 (92%)	184 (31%)<br>sICH: 1 (8%)	591 (100%)<br>sICH: 12 (100%)
**TNK (n=313)**			
**Any ICH (n=26)**			
**No OAC**	183 (59%)<br>Any ICH: 17 (65%)	87 (28%)<br>Any ICH: 5 (19%)	270 (86%)<br>Any ICH: 22 (85%)
**OAC**	27 (9%)<br>Any ICH: 3 (12%)	16 (5%)<br>Any ICH: 1 (4%)	43 (14%)<br>Any ICH: 4 (15%)
∑	210 (67%)<br>Any ICH: 20 (77%)	103 (33%)<br>Any ICH: 6 (23%)	313 (100%)<br>Any ICH: 26 (100%)
**sICH (n=8)**			
**No OAC**	183 (59%)<br>sICH: 6 (75%)	87 (28%)<br>sICH: 0 (0%)	270 (86%)<br>sICH: 6 (75%)
**OAC**	27 (9%)<br>sICH: 1 (13%)	16 (5%)<br>sICH: 1 (13%)	43 (14%)<br>sICH: 2 (25%)
∑	210 (67%)<br>sICH: 7 (88%)	103 (33%)<br>sICH: 1 (13%)	313 (100%)<br>sICH: 8 (100%)
**rt-PA (n=278)**			
**Any ICH (n=20)**			
**No OAC**	171 (62%)<br>Any ICH: 12 (60%)	65 (23%)<br>Any ICH: 4 (20%)	236 (85%)<br>Any ICH: 16 (80%)
**OAC**	26 (9%)<br>Any ICH: 3 (15%)	16 (6%)<br>Any ICH: 1 (5%)	42 (15%)<br>Any ICH: 4 (20%)
∑	197 (71%)<br>Any ICH: 15 (75%)	81 (29%)<br>Any ICH: 5 (25%)	278 (100%)<br>Any ICH: 20 (100%)
**sICH (n=4)**			
**No OAC**	171 (62%)<br>sICH: 2 (50%)	65 (23%)<br>sICH: 0 (0%)	236 (85%)<br>sICH: 2 (50%)
**OAC**	26 (9%)<br>sICH: 2 (50%)	16 (6%)<br>sICH: 0 (0%)	42 (15%)<br>sICH: 2 (50%)
∑	197 (71%)<br>sICH: 4 (100%)	81 (29%)<br>sICH: 0 (0%)	278 (100%)<br>sICH: 4 (100%)

Cross-tabulation of any ICH and sICH (SITS-MOST) stratified by IVT ≤4.5 h vs >4.5 h and OAC vs no OAC for overall cohort, TNK, and rt-PA. In each cell, the upper value refers to the corresponding patient row, whereas the lower value refers to the corresponding event row. Percentages in patient rows are calculated using the respective group total as denominator (overall, n=591; TNK, n=313; rt-PA, n=278); percentages in event rows are calculated using the total number of corresponding events within the respective group.

Abbreviations: ICH, intracranial hemorrhage; IVT, intravenous thrombolysis; sICH, symptomatic intracranial hemorrhage; OAC, oral anticoagulation.

Supplementary Table 5: Unadjusted and propensity score-based analyses of intracranial hemorrhage

Outcome and analysis	TNK	rt-PA	Risk difference (95% CI)	P value
Any ICH — unadjusted	26/313 (8%)	20/278 (7%)	—	0.61[1]
Any ICH — post-PSM	23/255 (9%)	19/255 (7%)	—	0.62[2]
Any ICH — PSM effect	—	—	0.0157 (−0.0305 to 0.0619)	0.51[3]
Any ICH — AIPTW ATT	—	—	0.0227 (−0.0168 to 0.0621)	0.26[4]
sICH (SITS-MOST) — unadjusted	8/313 (3%)	4/278 (1%)	—	0.34[1]
sICH (SITS-MOST) — post-PSM	8/255 (3%)	4/255 (2%)	—	0.34[2]
sICH (SITS-MOST) — PSM effect	—	—	0.0157 (−0.0086 to 0.0400)	0.21[3]
sICH (SITS-MOST) — AIPTW ATT	—	—	0.0154 (−0.0046 to 0.0355)	0.13[4]

Unadjusted analyses and AIPTW use the full cohort (TNK n=313; rt-PA n=278). PSM analyses use 255 matched pairs. Risk differences are TNK minus rt-PA on the probability scale; multiply by 100 to obtain percentage points. Positive values indicate higher risk under TNK. [1] Pearson chi-squared test without continuity correction. [2] Exact McNemar test. [3] Marginal risk difference from treatment-only logistic regression with pair-cluster robust variance; Wald confidence interval and test. [4] Normalized AIPTW ATT with a joint estimating-equation sandwich variance accounting for the propensity score and outcome models; Wald confidence interval and test.
The logistic propensity score model includes age, hypertension, CHA_2DS_2-VASc score and treatment within 4.5 h. The logistic outcome model includes prior antiplatelet therapy, baseline NIHSS, age and EVT. For ATT augmentation, the outcome model is fitted in rt-PA recipients and predicts outcomes under rt-PA for TNK recipients. No interactions are included.
Abbreviations: AIPTW, augmented inverse probability of treatment weighting; ATT, average treatment effect on the treated; CI, confidence interval; EVT, endovascular thrombectomy; ICH, intracranial hemorrhage; NIHSS, National Institutes of Health Stroke Scale; PSM, propensity score matching; sICH, symptomatic ICH.

Supplementary Table 6: Baseline characteristics and covariate balance before and after matching

Characteristic	Before PSM TNK n=313	Before PSM rt-PA n=278	SMD before	After PSM TNK n=255	After PSM rt-PA n=255	SMD after
Age, years*	75 (63-84)	77 (66-85)	0.159	78 (68-85)	77 (66-85)	0.071
Female sex	143/313 (46%)	135/278 (49%)	0.058	122/255 (48%)	124/255 (49%)	0.016
**Prior medical history; n (%)**						
Premorbid mRS; median (IQR)	1 (0-2)	1 (0-3)	0.116	1 (0-3)	1 (0-3)	0.038
Arterial hypertension*	221/313 (71%)	228/278 (82%)	0.271	206/255 (81%)	205/255 (80%)	0.009
CHA_2DS_2-VASc score*; median (IQR)	3 (2-5)	4 (2-5)	0.195	4 (3-5)	4 (2-5)	0.067
Any prior antiplatelet therapy	107/313 (34%)	99/278 (36%)	0.030	98/255 (38%)	89/255 (35%)	0.074
Oral anticoagulation	43/313 (14%)	42/278 (15%)	0.039	38/255 (15%)	38/255 (15%)	0.000
**Admission status**						
NIHSS on admission; median (IQR)	6 (3-11)	6 (4-11)	0.107	6 (4-11)	6 (4-11)	0.087
Symptom onset to door, min; median (IQR)†	85 (61-122); n=186	80 (56-124); n=174	0.035	80 (59.5-118); n=159	80 (57-124); n=157	0.060
Treatment within 4.5 h*; n (%)	210/313 (67%)	197/278 (71%)	0.082	186/255 (73%)	177/255 (69%)	0.076
**Stroke characteristics**						
Large vessel occlusion; n (%)	98/313 (31%)	97/278 (35%)	0.076	79/255 (31%)	89/255 (35%)	0.083
EVT among all patients; n (%)	71/313 (23%)	67/278 (24%)	0.033	54/255 (21%)	63/255 (25%)	0.083

Values are median (IQR) or count/denominator (%).
* Covariates included in the propensity score model. All four matching covariates have an absolute SMD <0.1 after matching. P values are omitted from the baseline balance table; SMDs quantify imbalance.
SMDs use a fixed pooled denominator from the full cohort: the square root of the average group sample variances for continuous variables, and $\sqrt{[p_1(1-p_1)+p_0(1-p_0)]/2}$ for binary variables.
Matching used ascending propensity score order, 1:1 nearest-neighbor matching without replacement on the logit scale, and a caliper of 0.060509945547 (0.2 SD of the full-cohort logit scores). This variant was selected after comparing matching orders; it was not the originally prespecified order.
† Restricted to recorded known symptom onset with an available interval; n is stated in each cell.
Any antiplatelet therapy includes dual and single antiplatelet therapy.
Abbreviations: DAPT, dual antiplatelet therapy; EVT, endovascular thrombectomy; IQR, interquartile range; LVO, large vessel occlusion; mRS, modified Rankin Scale; NIHSS, National Institutes of Health Stroke Scale; PSM, propensity score matching; SMD, standardized mean difference.

Supplementary Table 7: Primary workflow outcomes after propensity score matching

Procedure times, min; median (IQR)	TNK	rt-PA	Complete pairs	P value
Door to needle (TNK n=255; rt-PA n=255)	31 (22–49)	36 (26–55)	255	0.005
Door to imaging (TNK n=254; rt-PA n=251)	17 (12–23)	18 (14–23)	250	0.41
Imaging to needle (TNK n=254; rt-PA n=252)	12 (7–25)	16 (9–30)	251	0.020
Door to groin(TNK n=53; rt-PA n=63)	70 (63–83)	75 (66–99.5)	12	0.031
Door to recanalization (TNK n=49; rt-PA n=57)	109 (90–129)	118 (94–156)	10	0.76
DTN ≤30 min (TNK n=255; rt-PA n=255)	125 (49%)	101 (40%)	255	0.032

Continuous outcomes are median (IQR) among all patients with an available measurement in each group. Their p values use a paired Wilcoxon signed-rank test in complete pairs only (normal approximation, tie correction and continuity correction). Binary outcomes use the exact McNemar test.
Abbreviations: DTG, door to groin; DTI, door to imaging; DTN, door to needle; DTR, door to recanalization; EVT, endovascular thrombectomy; IQR, interquartile range; ITN, imaging to needle.

Supplementary Table 8: Clinical outcomes after propensity score matching

Outcome	TNK (n=255)	rt-PA (n=255)	P value
mRS at discharge, median (IQR)	3 (2-3)	3 (2-4)	0.18[1]
mRS distribution at discharge; n (%)			0.39[3]
mRS 0	28 (11%)	25 (10%)	
mRS 1	32 (13%)	33 (13%)	
mRS 2	58 (23%)	58 (23%)	
mRS 3	77 (30%)	66 (26%)	
mRS 4	29 (11%)	29 (11%)	
mRS 5	12 (5%)	25 (10%)	
mRS 6	19 (7%)	19 (7%)	
Excellent outcome at discharge (mRS 0–1); n (%)	60 (24%)	58 (23%)	0.91[2]
Favorable outcome at discharge (mRS 0–2); n (%)	118 (46%)	116 (45%)	0.92[2]
mRS 0–1 or return to premorbid mRS; n (%)	121 (47%)	112 (44%)	0.47[2]
mRS 0–2 or return to premorbid mRS; n (%)	171 (67%)	156 (61%)	0.18[2]
NIHSS at discharge, median (IQR)	2 (0.5-4); n=235	2 (0-5); n=235	0.11[1]
In-hospital mortality; n (%)	19 (7%)	19 (7%)	1.00[2]
Length of hospital stay, days, median (IQR)	5 (3-10)	6 (3-11)	0.59[1]
Discharge destination; n (%)			0.70[3]
Death	19 (7%)	19 (7%)	
Rehabilitation	61 (24%)	69 (27%)	
Nursing home	20 (8%)	25 (10%)	
Home	141 (55%)	132 (52%)	
Other clinic	14 (5%)	10 (4%)	
Stroke mimics; n (%)	7 (3%)	12 (5%)	0.33[2]

[1] Paired Wilcoxon signed-rank test. [2] Exact McNemar test. [3] Stuart-Maxwell test of marginal homogeneity.
Abbreviations: CI, confidence interval; IQR, interquartile range; mRS, modified Rankin Scale; NIHSS, National Institutes of Health Stroke Scale; OR, odds ratio.

Supplementary Figure 1: Adjusted predicted probabilities by treatment, stroke severity, and age.

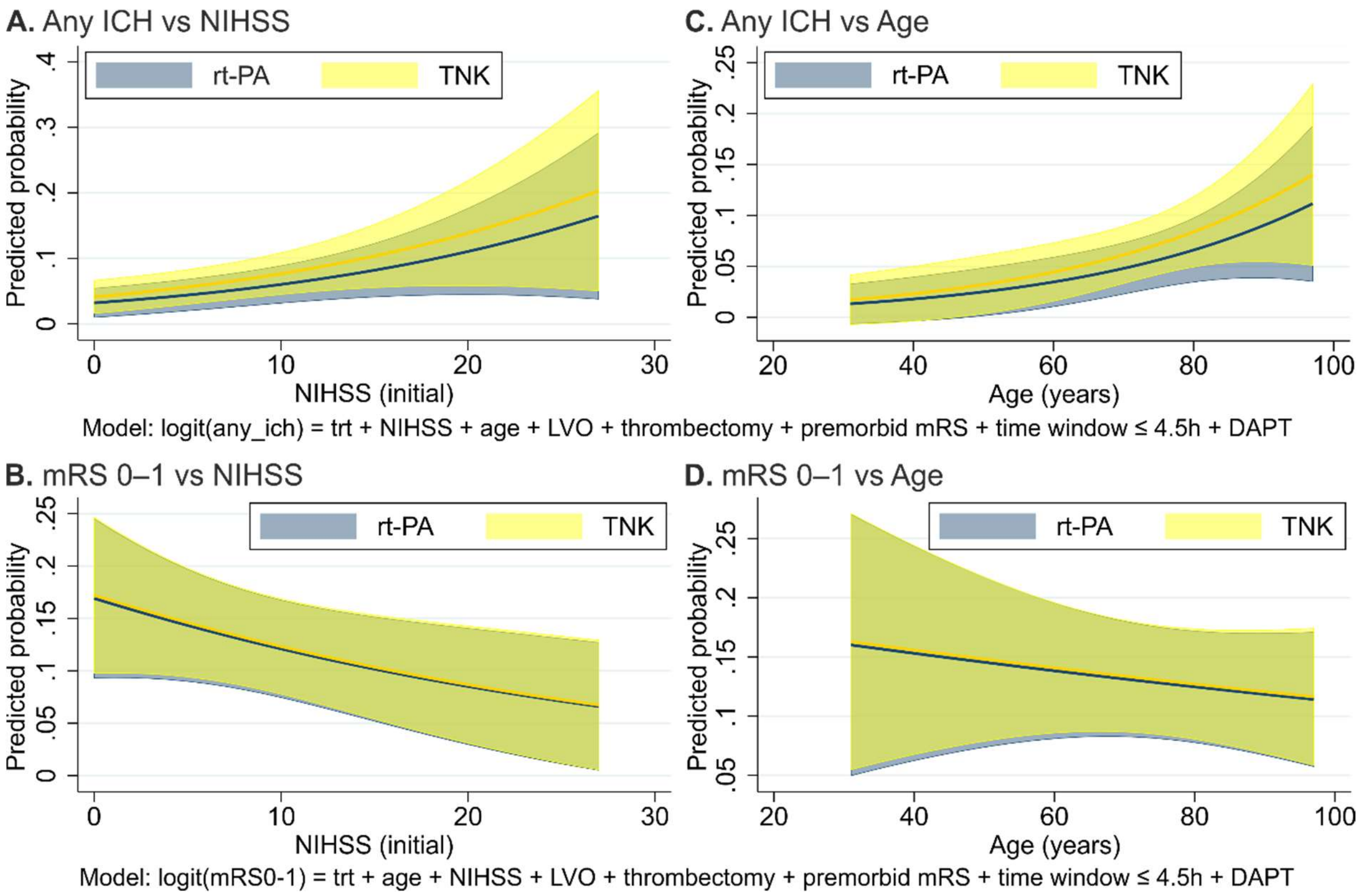

Adjusted predicted probabilities (95% CI) for any intracranial hemorrhage (A, C) and excellent outcome (mRS 0–1; B, D) according to baseline NIHSS (A, B) and age (C, D), for TNK and rt-PA. Estimates are based on multivariable logistic regression models as specified in each panel. Abbreviations: DAPT, dual antiplatelet therapy; ICH, intracranial hemorrhage; LVO, large vessel occlusion; mRS, modified Rankin Scale; NIHSS, National Institutes of Health Stroke Scale; trt, treatment.

Supplementary Figure 2: Adjusted odds ratios for any intracranial hemorrhage by subgroup.

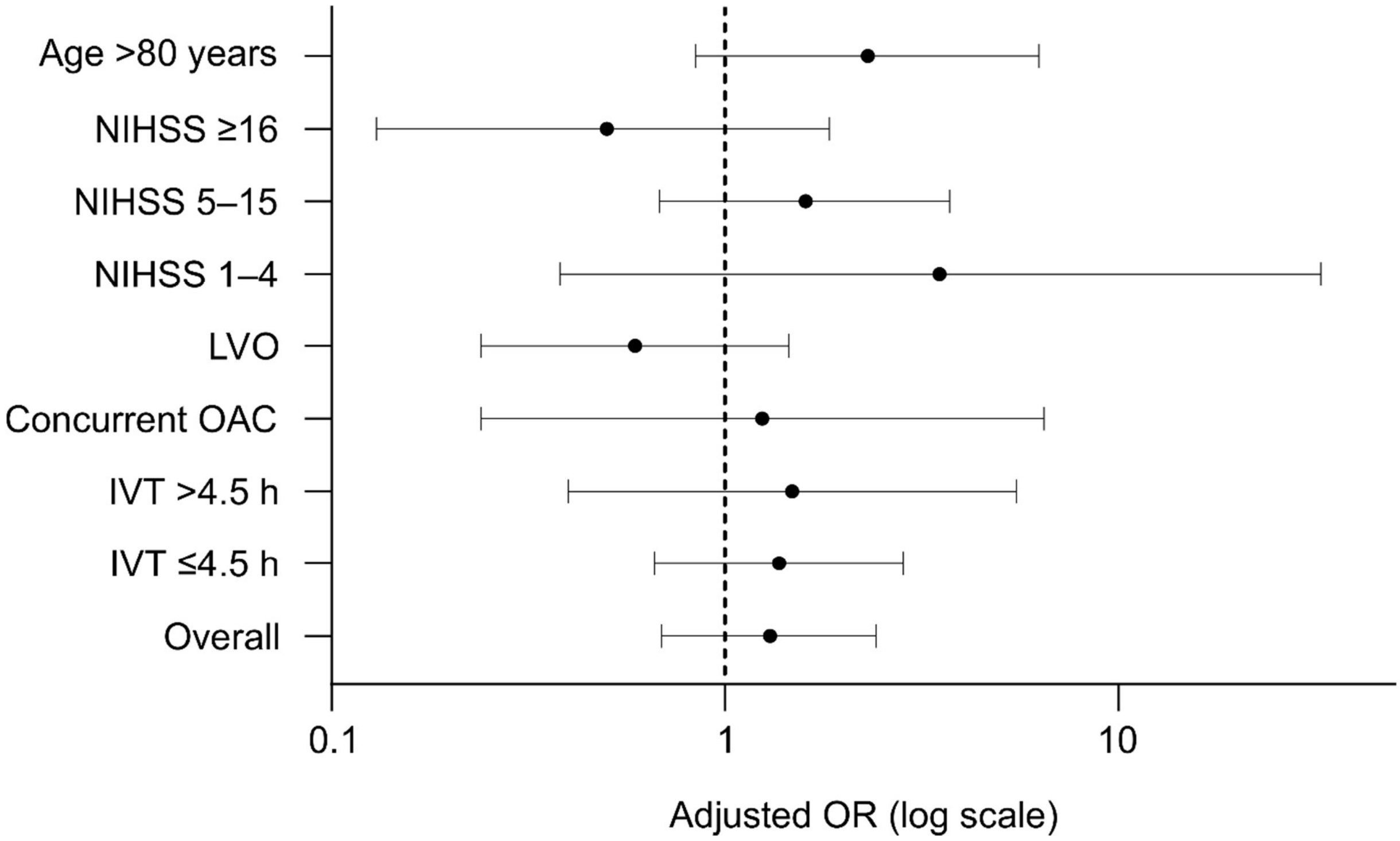

Forest plot of adjusted odds ratios with 95% confidence intervals for any intracranial hemorrhage comparing TNK versus rt-PA in the overall cohort and prespecified subgroups (treatment window ≤4.5 h vs. >4.5 h, oral anticoagulation, large vessel occlusion, baseline NIHSS 1–4/5–15/≥16, age >80 years). Estimates were obtained from multivariable logistic regression models; in subgroup analyses, the respective subgroup variable was omitted from the adjustment set. The dashed vertical line indicates an odds ratio of 1.0. Abbreviations: ICH, intracranial hemorrhage; LVO, large vessel occlusion; NIHSS, National Institutes of Health Stroke Scale; OAC, oral anticoagulation; OR, odds ratio; TW, time window.

Supplementary Figure 3: Covariate balance before and after propensity score matching.

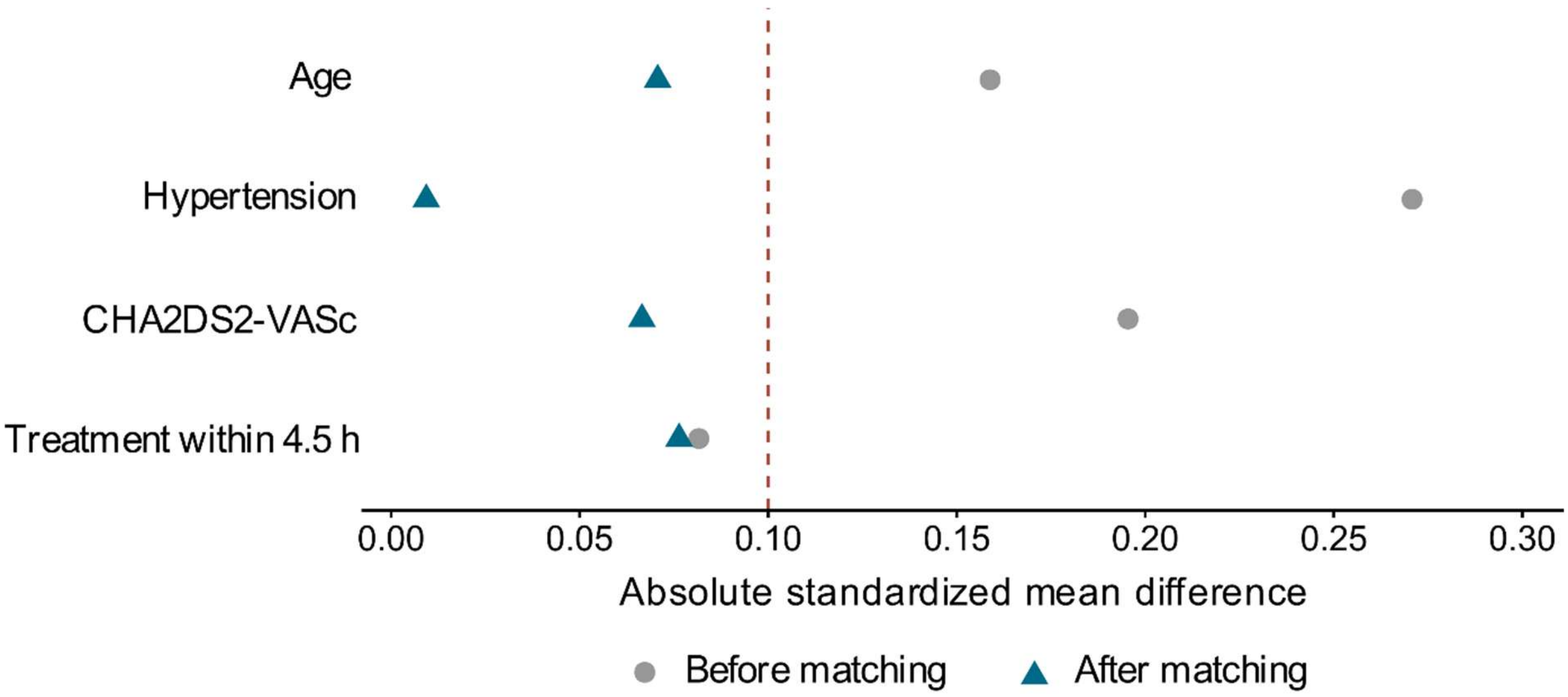

591 patients before matching and 255 matched pairs after matching. Love plot showing SMD for the four covariates included in the propensity score model before and after matching. The vertical dashed line indicates the prespecified threshold of SMD=0.1, with values below 0.1 considered indicative of adequate covariate balance. Abbreviations: SMD, standardized mean difference; PSM, propensity score matching.
